# COMPARING HEALTH CARE WORKFORCE IN NURSING HOMES BETWEEN TAIWAN AND THE UNITED STATES: A PILOT STUDY

**DOI:** 10.1093/geroni/igae098.3161

**Published:** 2024-12-31

**Authors:** Bianca Shieu, Ya-Wen Lee, John Harris

**Affiliations:** UT Health San Antonio, San Antonio, Texas, United States; Changhua Christian Hospital, Changhua, Changhua, Taiwan (Republic of China); Magee-Womens Research Institute, Pittsburgh, Pennsylvania, United States

## Abstract

Nursing homes (NHs) play a pivotal role in providing long-term care for elderly and chronically ill individuals. However, limited research has focused on understanding resident composition, staffing levels, and quality of care across different cultures. Therefore, this study aims to comprehensively compare the NH workforce between Taiwan and the United States, identifying improvement opportunities, enhancing staffing practices, and ultimately improving the lives of NH residents. This comparative analysis specifically examines two hospital-affiliated NHs in the US and three Christian-affiliated hospital-affiliated General-NHs in Taiwan. The first author conducted observations and interviews at two Hospital-affiliated NHs located in the Northeastern region of the United States, while the second author conducted observations and interviews at three Christian-based hospital-affiliated General NHs situated in Central Taiwan. Both authors spent several hours each day walking through the NHs, documenting their observations through field notes after each observation setting, conducting interviews with NH staff, and engaging with administrators to gather additional demographic information. We used Atlas.ti software for data analysis of our interviews. This study identified five distinct aspects warranting discussion due to their differences: ownership structure, NH resident demographics, medication administration protocols, staff training practices, and staffing shortages. In conclusion, the analysis of the healthcare workforce in NHs between Taiwan and the United States reveals critical insights into the challenges and opportunities faced by both nations. Additionally, this study contributes to a deeper understanding of NHs’ workforce dynamics, with implications for policy, practice, and resident well-being.

